# Remote Magnetic versus Manual Navigation for Radiofrequency Ablation of Paroxysmal Atrial Fibrillation: Long-Term, Controlled Data in a Large Cohort

**DOI:** 10.1155/2017/6323729

**Published:** 2017-03-13

**Authors:** Vikas Kataria, Benjamin Berte, Yves Vandekerckhove, Rene Tavernier, Mattias Duytschaever

**Affiliations:** ^1^Department of Cardiology, St. Jan Hospital Bruges, Ruddershove 10, 8000 Bruges, Belgium; ^2^University Hospital of Ghent, Ghent, Belgium

## Abstract

*Purpose.* We aimed to study long-term outcome after pulmonary vein isolation (PVI) guided by remote magnetic navigation (RMN) and provided comparative data to outcome after manual navigation (MAN).* Methods.* Three hundred thirty-six patients with symptomatic paroxysmal AF underwent PVI by irrigated point-by-point radiofrequency (RF) ablation (RMN, *n* = 114 versus MAN, *n* = 222). Patients were followed up with symptom guided rhythm monitoring for a period up to 43 months. The end point of the study was freedom from repeat ablation after a single procedure and without antiarrhythmic drug treatment (ADT).* Results.* At the end of follow-up (median 26.3 months), freedom from repeat ablation was comparable between RMN and MAN (70.9% versus 69.5%, *p* = 0.61). At repeat, mean number of reconnected veins was 2.4 ± 1.2 in RMN versus 2.6 ± 1.0 in MAN (*p* = 0.08). The majority of repeat procedures occurred during the first year (82.1% in RMN versus 78.5% in MAN; *p* = 0.74).* Conclusion.* On the long term (up to 3 years) and in a large cohort of patients with paroxysmal AF, RMN-guided PVI is as effective as MAN guided PVI. In both strategies the majority of repeat procedures occurred during the first year after index procedure.

## 1. Introduction

Percutaneous pulmonary vein isolation (PVI) has become an established treatment for patients with drug-refractory, paroxysmal atrial fibrillation (AF) [1]. Conventionally, PVI is performed by manual (MAN) point-by-point radiofrequency (RF) ablation. Ablation via remote navigation systems is an alternative strategy to perform point-by-point PVI. Various remote navigation systems available today can be divided into two categories: electromagnetic systems (*NIOBE™/EPOCH™*, Stereotaxis Inc., and GCI navigation system, Magnetics Inc.) and the electromechanical systems (Sensei, Hansen Medical Inc., and Amigo, Catheter Robotics Inc.). Most clinical data available is with remote magnetic navigation (RMN) via the* NIOBE/EPOCH* navigation system.

Potential benefits of the RMN include operator's comfort, reduced radiation exposure for both operator and patient, and improved safety of the procedure due to precision of catheter movement, soft tip, and the stability of the catheter. Stability of the catheter during ablation may lead to stable and effective lesions and hence better outcome.

Despite a lower PV isolation rate, single center observational studies suggest good midterm outcome at 6 to 12 months in RMN group compared to MAN group (freedom from atrial arrhythmia of 53.5 to 69% in RMN versus 55.5 to 68% in MAN) [2–7]. These studies, however, reflect early experience, short to midterm follow-up in a limited number of patients from a mixed population of paroxysmal and persistent AF. The HRS/EHRA/ECAS Expert Consensus Statement encourages reporting “long-term” outcome defined as a period of minimum 36 months [8].

The objective of the present study was to compare long-term outcome after RMN guided PVI in a large, contemporary cohort of patients with paroxysmal AF only and provide comparable data to MAN. The endpoint of the study was a repeat left atrial (LA) ablation procedure.

## 2. Methods

### 2.1. Study Population

All patients with symptomatic, drug-resistant, paroxysmal AF who underwent a first PVI via point-by-point RF ablation with an open-irrigated catheter between 1 January 2010 and 31 August 2012 were included in this* retrospective study*. The study population was followed up from the date of the index procedure till 31 August 2013 or till a repeat LA ablation procedure whichever was earlier. Paroxysmal AF was defined as recurrent AF (≥two episodes) that terminates spontaneously within 7 days. Episodes of AF of ≤48 hours' duration that are terminated with electrical or pharmacologic cardioversion were classified as paroxysmal AF [8].

The study population was divided into two groups according to the type of navigation during mapping and ablation: remote magnetic navigation (RMN group) or conventional manual navigation (MAN group). The endpoint of the study was a repeat LA ablation procedure.

Patients with nonparoxysmal AF, ablation with catheter other than open-irrigated catheter, and use of energy other than RF were excluded from the study.

The study was approved by the institutional ethical committee.

### 2.2. Ablation Procedure

Procedures were performed by two principal operators (MD, RT). All ablation procedures were performed under therapeutic anticoagulation. A 6F decapolar catheter was positioned in the coronary sinus (CS). Immediately prior to transseptal puncture an intravenous loading dose of 10,000 IU heparin was administered. A double transseptal puncture was performed to position two 8F nonsteerable sheaths (SL0, St. Jude Medical, St. Paul, MN, USA) in the LA. After puncture, a continuous infusion of heparin was started to maintain an activated clotting time (ACT) above 300 seconds throughout the procedure. A 3.5 mm open-irrigated tip ablation catheter (Navistar Thermocool or Navistar Thermocool Smart-touch, Biosense Webster, Diamond Bar, CA, USA) was used for mapping and ablation in MAN group, and an open 3.5 mm irrigated tip ablation catheter (Navistar RMT Thermocool, Biosense Webster, Diamond Bar, CA, USA) in the RMN group. Three-dimensional anatomy of the LA was reconstructed using CARTO-3 electroanatomical system (Biosense Webster, Diamond Bar, CA, USA).

A steerable multipolar circular mapping catheter (Lasso, Biosense Webster) was placed ≈0.5 cm within the PV to record baseline electrograms during sinus rhythm and differential pacing. The ablation procedure consisted of encircling the ipsilateral PVs by a continuous circular lesion which was created by point-by-point RF applications (30 to 60 seconds) with generator settings of 20–35 W, 20 cc/min flow rate, and 48°C cut-off temperature. The endpoint for ablation was LA-PV entry block with elimination of the PV potentials during sinus rhythm and differential pacing. If PVI was not obtained after encircling the ipsilateral veins, residual conduction gaps at the circumference were identified by the aid of the circular mapping catheter and subsequently closed by selective radiofrequency lesions. PVI was reassessed after 60 minutes. Adenosine was used at the discretion of the operator.

### 2.3. Remote Magnetic Navigation

The remote magnetic navigation system used to control catheter navigation in the present study consists of 2 independent but communicating components:* the NIOBE/EPOCH (Stereotaxis, St. Louis MO, USA) magnetic navigation system* and an electroanatomical mapping system (CARTO-RMT, Biosense Webster, Inc., Diamond Bar, CA, USA). The RMN system uses 2 large magnets positioned on either side of the procedure table to generate a composite magnetic field for directional catheter navigation. The magnetic tip of the ablation catheter (Navistar RMT ThermoCool, Biosense Webster, Diamond Bar, CA, USA) aligns itself with the direction of the externally controlled magnetic field to enable it to be steered effectively. By changing the orientation of the outer magnets relative to each other, the orientation of the magnetic field changes and thereby leads to deflection of the catheter. The navigation of the catheter is guided by the superimposed 3-dimensional anatomical image and the fluoroscopic image.

The CARTO-RMT electroanatomical system is similar to the standard CARTO system except that it is able to localize the ablation catheter without interference from the RMN system's magnetic field.

### 2.4. Follow-Up

After the procedure, patients were anticoagulated by subcutaneous low-molecular weight heparin, or oral anticoagulation therapy (OAT). Antiarrhythmic drug treatment (ADT) was reinstituted in all patients. After a 3-month blanking period, ADT (except for beta-blocking agents) were invariably stopped. All patients underwent follow-up (questionnaire, physical examination, and ECG) at scheduled (after 1 month, at 6 months, and then every 6 months during the follow-up) and unscheduled visits (if symptoms). Patients in both the groups were followed up by the two same physicians (MD, RT). In case of symptoms the related arrhythmia was documented either by repeated ECG, Holter monitoring (1 to 7-day), or event recording. In the event of recurrence (asymptomatic or symptomatic), repeat ablation was strongly advised.

### 2.5. Data Collection

The study population was followed up from the date of the index procedure till 31 August 2013 (therefore the minimum follow-up period was ≥ 1 year) or till a repeat LA ablation procedure whichever was earlier. The endpoint of the study was freedom from repeat LA ablation procedure following the 3-month blanking period through a minimum of 12 months follow-up from the date of the ablation procedure in the absence of Class I and III ADT.

Data related to patient characteristics, procedural characteristics, and procedural outcomes in the two groups (RMN and MAN) was collected from our institutional database. In the event of a repeat ablation procedure, data regarding presence of reconnected PVs, number or reconnected PVs, and their distribution was collected.

Major complication was defined as any complication that results in permanent injury or death, requires intervention for treatment, or prolongs hospitalization [8].

### 2.6. Statistical Analysis

Data are presented as mean ± SD or as percentages. Differences between groups were determined by *t*-test and Fisher's exact test. Kaplan-Meir survival curves are used for comparing freedom from repeat procedure between the two groups. Uni- and multivariate analysis was performed using Cox-regression analysis. A *p* value of less than 0.05 was considered significant. IBM SPSS version 20.0 was used for statistical analysis.

## 3. Results

### 3.1. Patient Characteristics

Out of 414 cases of first PVI procedure for paroxysmal AF performed at our institution between 1 January 2010 and 31 August 2012, 336 were found eligible for the study. Of these 336 patients 222 were performed by MAN (Navistar Thermocool, *n* = 203, and Navistar Thermocool Smart-Touch, *n* = 19) and 114 by RMN.

Baseline clinical characteristics were not different between both groups (Table 1). Mean age of the patients in RMN group was 61.3 ± 9.6 years and in the MAN group was 59.9 ± 16.2 years with 65.7% and 61.2% males in respective groups.

### 3.2. Procedural Data and Acute Procedural Success

Complete pulmonary vein isolation was achieved in all the patients in both the groups. To test for operator bias, RMN versus MAN ablations done by the same operator were compared and no difference was found. Mean duration of procedure was significantly higher in RMN group than MAN group (291.3 ± 81.2 and 231.4 ± 71.2 minutes, resp., *p* < 0.0001).* Mean RF time for RMT (3795 ± 1322 sec) was significantly higher than RF time for MAN (3109 ± 1188 sec, p* < 0.0001). Mean fluoroscopy duration was 35.2 ± 18.5 minutes in RMN group and 39.9 ± 16.4 minutes in MAN group (*p* = 0.017).

### 3.3. Safety of Ablation

Major complications were similar in RMN and MAN groups (1.75% versus 1.8%, resp., *p* = 1.0). In the RMN group, 1 patient developed tamponade (requiring percutaneous drainage) due to inadvertent needle puncture of the LA wall and one patient developed TIA. In MAN group, 3 out of 222 patients developed tamponade (requiring percutaneous drainage) and one developed a vascular access site complication requiring surgical intervention. Minor complications in both groups consisted of minor bleeding from the vascular access site (2.6% versus 3.1%, resp., *p* = 0.54).

### 3.4. Long-Term Outcome after Ablation

**Figure 1:**
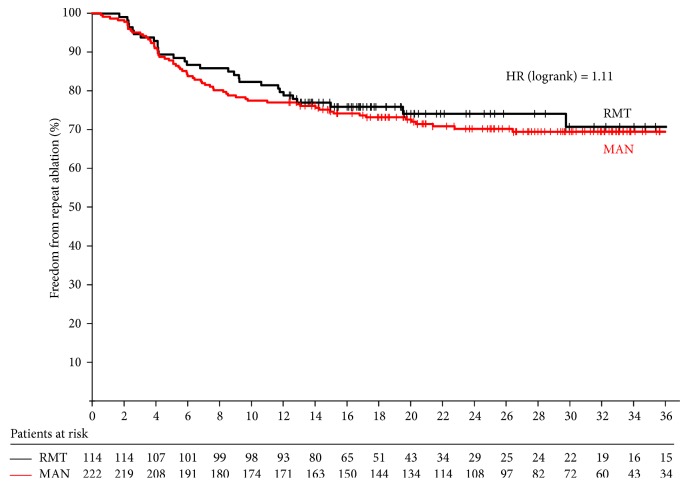
Survival curves showing freedom from repeat ablation in both groups.

The survival curve is shown in Figure 1. Longest follow-up was 43 months with a median of 26.3 months and a mean of 27.2 ± 8.8 months.* The number of patients lost to follow-up at 1, 2, and 3 years was 0, 35, and 45, respectively*. At 43 months of follow-up, freedom from repeat procedure without ADT was 70.9% in RMN group and 69.5% in MAN group (*p* value = 0.61).

In the RMN group 29 (25.4%) patients had a repeat LA ablation procedure, whereas in MAN group 65 (29.2%) patients had a repeat ablative procedure at 43 months of follow-up after the index procedure. Majority of the repeat ablations occurred in the first year following the index procedure (82.1% in RMN versus 78.5% in MAN; *p* = 0.74). Mean time to repeat procedure was 240.1 ± 185.5 days in RMN group and 231.8 ± 181 days in MAN group (*p* = 0.69) (Table 2). No clinical or procedural factors were associated with AF recurrence. No clinical or procedural parameter was predictive for early (≤1 year) or late recurrences (>1 year).

### 3.5. Repeat Ablation Characteristics

Mean number of reconnected veins was 2.4 ± 1.2 in RMN group and 2.6 ± 1.0 in MAN group (*p* = 0.08). The distribution of reconnected veins was comparable in two groups (Table 2). Two patients in each group underwent a repeat procedure for AT and had no PV reconnection.

## 4. Discussion

### 4.1. Main Findings

In the present study long-term outcome of RMN was compared to MAN for PV isolation of paroxysmal AF. At 43 months* (median 26.3 months or *≈*2 years)* of follow-up and without ADT, irrigated RF ablation with RMN is comparable to MAN for freedom from repeat ablation. The follow-up duration of 43 months is in accordance with the recommended duration while reporting long-term results [8].

### 4.2. Repeat Ablation Procedure as an Efficacy Endpoint

Freedom from symptomatic and/or asymptomatic AF, “AF control” defined as greater than 90% reduction of AF burden, proportion of patients free of AF in a given period of time, and an ECG or Holter monitor administered at certain period of time following the ablation procedure and improved quality of life have been the conventional endpoints in the studies related to catheter ablation of AF [8–10]. When comparing technologies/techniques these endpoints have obvious limitations like discrepancies in the definition of recurrence, recurrence rate being heavily dependent on the definition, the method of follow-up, and definition of blanking period. In retrospective studies, especially, these endpoints are prone to bias.

Freedom from repeat ablation as an endpoint is an economical and completely objective hard endpoint. Such an endpoint in a prospective study would be subjected to selection bias (unless blinded). But in a retrospective study, like the present one, it is a strong endpoint free from any bias, to compare the efficacy of two techniques (rather than to evaluate the effectiveness of single method alone).

### 4.3. Prior Comparative Studies on the Efficacy of RMN Guided PV Isolation

Several studies have reported similar short and midterm outcome data on RMN versus MAN guided PVI using irrigated point-by-point RF ablation. Shinsuke Miyazaki et al. compared these two methods in 30 consecutive patients for 12 months and reported freedom from atrial tachyarrhythmia of 69.0% in magnetic and 61.8% in manual group (*p* = 0.961) [4]. Arash Arya et al. studied 356 patients (remote navigation, *n* = 70, and manual navigation, *n* = 286) and reported freedom from AF at 6 months of 60.5% and 68.2% (*p* = 0.36) in the two groups, respectively [6]. Other studies and two recent meta-analysis also found the two navigation systems to be comparable in preventing the recurrence of AF and atrial tachyarrhythmia [7, 9, 10]. These studies provided only short-term (6–12 months) comparison of the two navigation methods, often in a mixed and/or small population of paroxysmal and persistent AF.

The present study compared the two strategies in 336 paroxysmal AF patients over 43 months' duration and showed that the freedom from repeat ablation after PVI in RMN group is equal to the MAN group. Of course the data in the present study does not reflect the actual recurrence rate. Similar efficacy results with the soft and flexible ablation catheter in RMN group may be a reflection of stable catheter contact at a single point for a longer period of time, hence resulting in a good force-time integral (FTI) and thus effective lesions [11]. In addition, the similar number of reconnected veins during repeat procedure in both the groups reflects equal quality of lesions.

### 4.4. Periprocedural Data on Efficiency and Safety

Higher procedural time in the RMN group may be attributed to the additional time required to set up the navigational system and time required for moving to and from RMN room to the operation table for placing the circular mapping catheter into different veins. Similar results of higher procedural time during RMN were also reported by Miyazaki et al. and Arya et al. and the two meta-analyses [4, 6, 9, 10].

Fluoroscopic duration in this study was lower in the RMN group. This could be attributed to increased operator's confidence during catheter manipulation due to flexible ablation catheter in the RMN group, better precision of catheter movement, and stable catheter position. Similar result of lower fluoroscopy time was observed by Arya et al. and the two meta-analyses [6, 9, 10].

Complication rate was similar in both the groups. Lower complication rate in RMN than MAN has been reported by Choi MS et al. and in a recent meta-analysis [5, 10]. Similar rate of major complication in both groups in the present study may be attributed to the fact that these complications were “preablation” (transseptal puncture related tamponade) and hence were not related to the method of navigation or the type of ablation catheter.

### 4.5. Limitations

The study being a retrospective nonrandomized study has its inherent limitations.* The study population was followed up from the date of the index procedure till 31 August 2013 or till a repeat LA ablation procedure (in our department) whichever was earlier. This study design has two limitations: (1) follow-up ranged from 12 to 43 months, and (2) it does not take into account the likelihood of patients undergoing repeat ablation in another center (actual “lost to follow-up” patients). However, the likelihood of repeat ablation in another center is estimated to be limited and the same for both groups*.

*Despite similar baseline characteristics and similar use of ablation energy (irrigated RF) and ablation settings, groups (RMN* versus* MAN) could differ in unknown or unmeasured variables (like obesity and renal function)*.

Holter monitoring or event recording was only performed in the event of symptoms. More aggressive monitoring would have revealed asymptomatic recurrences. On the other hand, symptoms are the driver for an AF patient to undergo ablation and, for the purpose of comparison, freedom from repeat ablation is a better endpoint, as discussed earlier.

*The RF settings used in the current study (for both RMN and MAN) might differ from those settings currently applied in RF ablation strategies*. Moreover, increased use of ablation catheter with “contact-force” sensing technology in MAN group might yield better results than RMN.

## 5. Conclusions

*At 43 months (median 26.3 months or *≈*2 years) of follow-up, for irrigated point-by-point RF ablation, RMN guided ablation is comparable to MAN for freedom from repeat ablation*. Majority of repeat procedures in both the groups occurred during the first year after index procedure. This data, together with its reported advantageous safety profile, further strengthens the potential role of RMN as a viable alternative in the ablation of paroxysmal AF.

## Figures and Tables

**Table 1:** Baseline clinical characteristics in two groups.

Characteristic	RMN (*n* = 114)	MAN (*n* = 222)	*p* value
Paroxysmal AF, *n* (%)	114 (100%)	222 (100%)	1.0
Age, years	61.3 ± 9.6	59.8 ± 16.2	0.3
Males, *n* (%)	75 (65.7%)	136 (61.2%)	0.47
CHADS_2_ Score	0.4 ± 1.3	0.4 ± 0.6	1.0
CHA_2_DS_2_-VASc Score	1.4 ± 1.3	1.3 ± 1.2	0.49
Hypertension, *n* (%)	35 (31%)	60 (27%)	0.52
Diabetes, *n* (%)	11 (9.6%)	17 (7.6%)	0.53
Structural heart disease, *n* (%)	4 (3.5%)	9 (4.05%)	1.0
LA diameter (PLAX view), mm	40.7 ± 4.7	39.6 ± 5.3	0.06

**Table 2:** Details of repeat procedures.

	RMN (*n* = 29)	MAN (*n* = 65)	*p* value
Time to repeat procedure, days	240.1 ± 185.5	231.8 ± 181	0.08
Repeat procedure at 1 year, *n* (%)	24 (82.7%)	51 (78.4%)	0.74
Mean number of reconnected veins	2.4 ± 1.2	2.6 ± 1.0	0.07
Total number of reconnected veins, *n*	68/114 PVs	164/253 PVs	0.65
Reconnection of RSPV, *n*	15/68	39/164	0.75
Reconnection of RIPV, *n*	23/68	43/164	0.56
Reconnection of LSPV, *n*	13/68	37/164	0.51
Reconnection of LIPV, *n*	15/68	38/164	0.75
Reconnection of LCPV	2/68	7/164	NA
